# Distinctive features of bimanual coordination in idiopathic normal pressure hydrocephalus

**DOI:** 10.1007/s00701-024-06363-w

**Published:** 2024-11-28

**Authors:** Takuma Umemori, Kazushige Kobayashi, Ryo Watanabe, Takahiro Higuchi

**Affiliations:** 1 Department of Health Promotion Science, Tokyo Metropolitan University, 1-1 Minami-Ohsawa, Hachioji, Tokyo, 192-0397 Japan; 2 Department of Rehabilitation, The Jikei University Katsushika Medical Center, 7-18-5 Aoto, Katsushika-ku, Tokyo, 125-8506 Japan; 3 The Jikei University School of Medicine, 3-19-18 Nishishimbashi, Minato-ku, Tokyo, 105-8471 Japan; 4 Nomura Hospital, 8-3-6 Shimorenjaku, Mitaka-shi, Tokyo, 181-8503 Japan; 5 Faculty of Sports Humanities and Applied Social Science, National Institute of Fitness and Sports in Kanoya, 1 Shiromizu, Kanoya, Kagoshima, 891-2393 Japan; 6 Japan Society for the Promotion of Science, 5-3-1 Kojimachi, Chiyoda-ku, Tokyo, 102-0083 Japan

**Keywords:** Idiopathic normal pressure hydrocephalus, Bimanual coordination, Finger tapping task, Occupational therapy

## Abstract

**Background:**

Idiopathic normal pressure hydrocephalus (iNPH) is characterized by cerebrospinal fluid circulation disorders, and presents as gait and balance disturbances similar to those observed in other incurable neurological diseases. Although previous studies have reported deficits in bimanual coordination among patients with iNPH, these potential pathological characteristics have not received much attention to date. This study investigated the temporal characteristics of a bimanual finger-tapping task in patients with iNPH, focusing on within- and between-hand coordination.

**Methods:**

Study participants comprised three groups: patients with iNPH (*N* = 19, mean age = 76.9 ± 5.6 years), older adults (*N* = 19, 76.4 **±** 5.3 years), and younger adults (*N* = 13, 32.2 **±** 8.5 years). Participants performed a bimanual finger-tapping task under five conditions that manipulated the temporal differences between the two taps.

**Results:**

The iNPH group exhibited significantly greater errors in both within- and between-hand coordination tasks compared to the other two groups.

**Conclusion:**

These results suggest that assessing temporal errors in bimanual coordination tasks, particularly within-hand coordination, may be useful for uncovering pathological characteristics specific to iNPH.

## Introduction

Idiopathic normal pressure hydrocephalus (iNPH) is a clinical syndrome characterized by ventricular dilation caused by an impairment in cerebrospinal fluid circulation [21, 24]. Treatment typically involves surgical intervention, such as spinal fluid shunting, which can significantly improve clinical symptoms and the quality of life of patients. However, because the symptoms of iNPH are similar to those of other dementias and neurological disorders [27], the disease is often misdiagnosed and left untreated without detailed examination [41]. For example, gait disorders in iNPH can manifest as a small-step, magnet gait, and wide-base gait [36, 37], whereas balance disorders are characterized by instability in a standing posture with legs in line [16]. It has been suggested that gait disturbance in iNPH may be related to lesions in the striatum and the corticospinal tract [6, 12, 25], and that the foci responsible for balance disturbance may involve both central and peripheral systems [1, 20, 34]. However, such gait and balance characteristics associated with iNPH may be overlooked given their prevalence in other types of individuals, such as older adults at a high risk of falling [5, 10, 13, 44], individuals with Parkinson’s disease [39], and those experiencing progressive supranuclear palsy [31]. Moreover, age-related cognitive decline can also influence the gait and balance impairments observed in patients with iNPH [26], complicating the attribution of these symptoms solely to iNPH. It is therefore necessary to develop diagnostic tasks that can accurately identify iNPH-specific pathological features.

Previous studies suggest that developing a behavioral task to assess the functions of the supplementary motor area would be advantageous. For example, reduced cerebral blood flow in the frontal lobe, particularly in the brain areas involved in motor control, has been reported to be an indicator of the unique pathological characteristics of iNPH [18, 22, 23]. Further, diminished blood flow in the supplementary motor area, which is located just below the higher arcuate region, has been observed across all stages of the disease [38]. Iseki et al. (2012) evaluated patients with iNPH and healthy older adults using the Frontal Assessment Battery (FAB), which encompasses both cognitive and motor tasks that engage frontal lobe functions [8]. Their findings showed that, compared to healthy older adults, patients with iNPH exhibited declines in specific test items: “programming”, assessed depending on whether Luria’s motor series (fist-palm-edge) would be performed in the correct order, and “mental flexibility”, evaluated through spontaneous word production tasks [15]. These assessments, which are indicative of functionality in higher motor areas, including the supplementary motor area, underscored the potential role of these regions in iNPH pathology [4, 40]. Consequently, these findings suggest that the development of a behavioral task with which the functions of the supplementary motor can be assessed is likely to be beneficial in delineating the specific pathological characteristics of iNPH.

Several previous studies have shown that a behavioral task involving bimanual coordination of the hands is a candidate for assessing neurological function and motor control [35, 43]. These studies implemented a bimanual finger-tapping task, requiring participants to tap buttons with their left and right index fingers in synchronization with auditory stimuli. Serrien et al. conducted experiments with younger adult participants under two tapping conditions: simultaneous and alternate [35]. Under the simultaneous condition, the index fingers of both hands were tapped in synchrony with auditory cues, whereas under the alternate condition, one finger was tapped in synchrony with auditory cues, while the other finger was tapped in silence. The authors employed high-frequency repetitive transcranial magnetic stimulation (rTMS) targeting the supplementary motor area (SMA) during the execution of the bimanual task to elucidate the functional contribution of the SMA to bimanual coordination. The findings indicated that the timing accuracy was degraded by applying high-frequency rTMS to the SMA, especially under the alternate condition. This deterioration in performance suggests that asynchronous bimanual coordination poses significant challenges for patients with iNPH who exhibit impaired blood flow to the SMA.

In a recent study, we assessed patients with iNPH and obtained similar findings to those of Serrien et al. [35, 43]. In this study [43], we used a finger-to-thumb tapping task under three tapping conditions: unimanual tapping, simultaneous bimanual tapping, and alternate bimanual tapping. The results showed that patients with iNPH exhibited significantly decreased accuracy and increased variability in tapping performance only under the alternate condition. The findings across both studies suggest that dysfunction in the SMA may manifest as impaired bimanual coordination when performed in an asynchronous manner. This assertion is supported by the established role of the SMA in controlling the temporal sequencing of movements [42].

The aim of the present study was to provide additional evidence supporting the hypothesis that impairments in the temporal aspects of bimanual coordination are distinctive pathological characteristic features of iNPH. To the end, we designed experiments to assess whether impaired bimanual finger-tapping, in terms of temporal coordination in patients with iNPH as reported in Umemori et al. [43], could be attributed solely to factors unique to iNPH. In Umemori et al., the frequency of auditory stimuli varied among the tapping conditions: 1 Hz for the unimanual and simultaneous conditions, and 0.5 Hz for the alternate condition [43]. This adjustment was required in order to set the tapping interval to 1 Hz across all conditions. Consequently, under the alternate condition, one finger tapped without concurrent auditory cues, potentially increasing task difficulty. It is therefore possible that the increased challenge under the alternate condition might explain the difficulties of patients with iNPH in performing the tapping task, separate from any disease-specific motor control deficits.

To reject this possibility, the present study tasked participants with performing a bimanual finger-tapping pattern with a unique tapping frequency, referred to here as “the gallop pattern”. In this pattern, tapping is non-isochronous, creating slight discrepancies between the taps of both index fingers, specifically at intervals of 250 or 500 ms [45]. Unlike the tapping under the alternate condition where taps might be perceived individually, taps under the gallop condition were perceived as a group [3]. For this experiment, we standardized the within-hand lag at a slow tempo (0.5 Hz) across all conditions, while varying the between-hand lag. Furthermore, all of the taps were synchronized with auditory cues. We examined whether, under these conditions, patients with iNPH would still exhibit difficulties in performing the task, especially in the alternat condition.

## Methods

### Participants

Nineteen patients with iNPH (iNPH group, mean age = 76.9 ± 5.6 years, 10 males and nine females) were recruited from the Department of Neurosurgery and Rehabilitation at the Jikei University Katsushika Medical Center. Patients were diagnosed with probable iNPH based on positive outcomes from a subarachnoid cerebrospinal fluid (CSF) tap test according to the iNPH diagnostic criteria [24]. The CSF tap test is the most common test for predicting clinical improvement post-shunting and involves the removal of 30–50 ml CSF [11]. This test is typically effective for ameliorating the clinical triad of symptoms associated with iNPH. All patients were right-handed.

The inclusion criteria for probable iNPH, as proposed in the national iNPH guidelines [24], were as follows: (1) symptomatic onset at the age of 60 years or older; (2) ventricular dilation detected on magnetic resonance imaging (Evans Index > 0.3); (3) presence of more than one symptom from the clinical triad, i.e., gait disturbance, cognitive impairment, and urinary incontinence; (4) the aforementioned clinical symptoms cannot be completely explained by other neurological or non-neurological diseases; (5) absence of preceding diseases that could cause ventricular dilation, such as subarachnoid hemorrhage, meningitis, head injury, congenital/developmental hydrocephalus, and aqueductal stenosis; (6) CSF pressure at or below 200 mmH_2_O with a normal CSF content; (7) two investigational features: (a) neuroimaging evidence of narrowing of the sulci and subarachnoid space over the high-convexity/midline surface (DESH), associated with gait disturbances such as small stride, shuffling, instability during walking, and increase in instability on turning, (b) improvement of symptoms following a CSF tap test.

Nineteen older healthy adults (older group, mean age = 76.4 ± 5.3 years, 12 males and seven females) and 13 younger healthy adults (younger group, mean age = 32.2 ± 8.5 years, six males and seven females) also participated in the experiment as control participants. Older adults were recruited from individuals registered for participation in experiments conducted by the Perception and Action Laboratory at Tokyo Metropolitan University. Younger adults were recruited from the medical staff of the center and students at Tokyo Metropolitan University. The exclusion criteria for all participant groups included one or more of the following: (1) difficulties in understanding and performing tasks; (2) left-handedness; (3) hearing-impairment; (4) presence of fractures or paresis in the upper extremities; or (5) presence of aphasia, apraxia, or agnosia.

Ethics approval for this study was obtained from the Ethics Committee of The Jikei University School of Medicine (Approval No.: 30–352 9373) and Tokyo Metropolitan University (Approval No.: H5-126). Prior to participation, all participants received a written explanation describing the purpose, methods, contents, voluntary nature of participation in this study, and protection of personal information. Informed consent was then obtained from participants after these disclosures. In conducting the research, the handling of personal information and all other aspects of the study adhered strictly to the Personal Information Protection Law and were conducted in accordance with the principles outlined in the Declaration of Helsinki.

### Apparatus

**Fig. 1:**
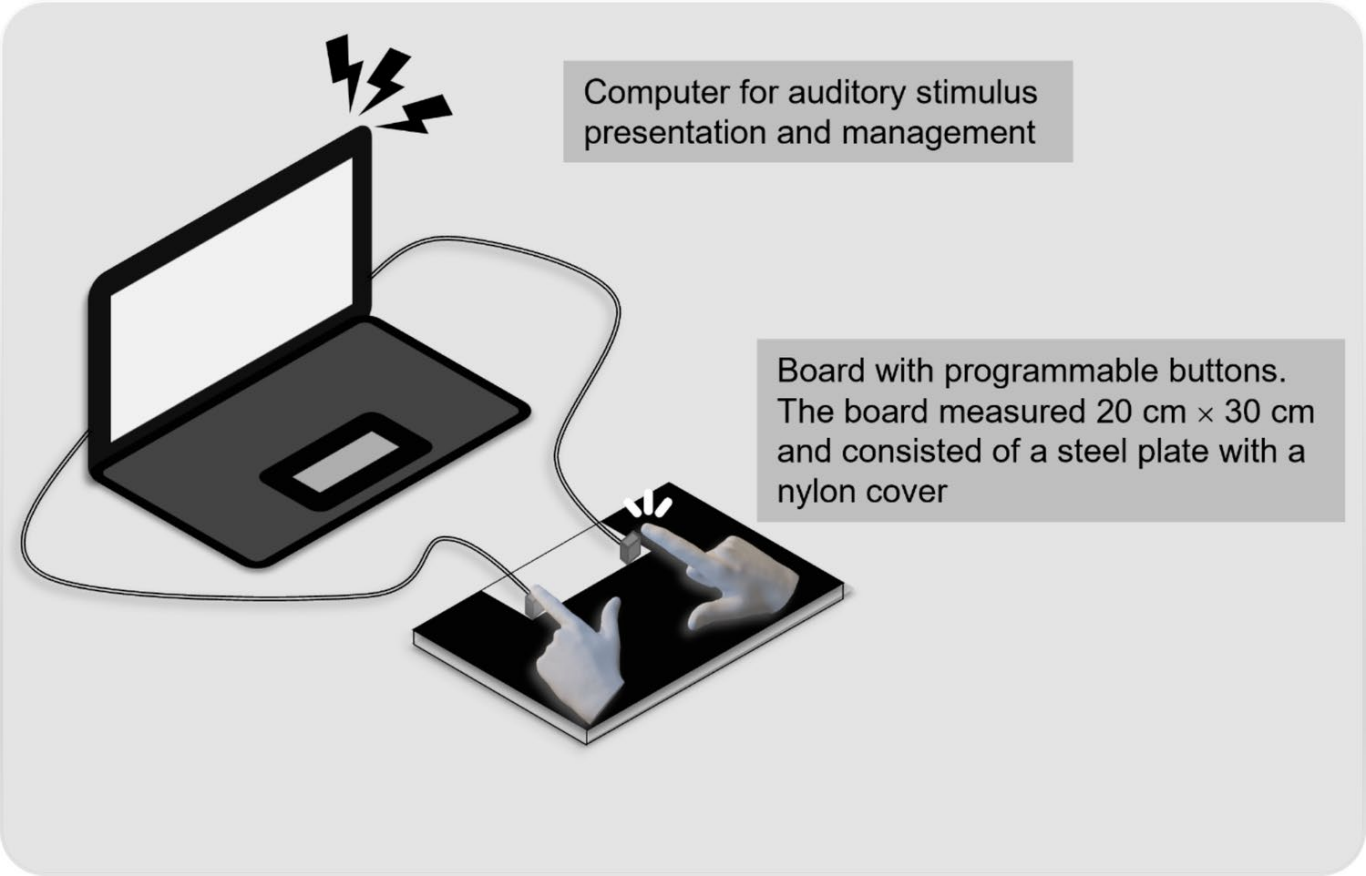
Experimental apparatus

Presentation of auditory stimuli and recording of tapping responses were managed using PsychoPy v2022.2.2 [30], which was installed on a laptop personal computer (Mouse Computer, DESKTOP-VECLM3L, operating on Windows 11, 64-bit, with a refresh rate of 240 Hz and sound output of 24 bit, 48000 Hz). Two HID-compliant programmable single buttons (NE Fashion01, Guangzhou, China) were mounted on a 20 cm$\:\times\:$ 30 cm steel plate and covered with a nylon sheet. The buttons, which were positioned such that the distance between the two index fingers of a participant was 14 cm, were connected to the computer (Fig. 1). Five distinct auditory stimuli were used in the experiment. The stimuli consisted of two identical sounds separated by varying intervals, each with a frequency of 440 Hz (duration 100 ms). The intervals were set as follows: 0 ms (in-phase, simultaneous tapping), 250 ms, 500 ms, 750 ms (constituting the gallop pattern), and 1000 ms (anti-phase, alternate tapping). The programmable buttons used for recording responses had a temporal resolution of 240 Hz.

The distance between the two keys is 114 mm (based on a key pitch of 19 mm, with 6 keys open). The space between the buttons is large enough for an adult male to place both hands on.

### Task and procedure

Participants were seated in a comfortable chair at a table and instructed to perform a bimanual finger-tapping task. The task involved pressing two separate buttons with their left and right index fingers, respectively, in synchrony with the two auditory stimuli. The task comprised five tapping conditions: simultaneous, gallop (250 ms), gallop (500 ms), gallop (750 ms), and alternate. Under the simultaneous condition, the two auditory stimuli were presented simultaneously, resulting in in-phase tapping. Under the three gallop conditions, time interval of the two auditory stimuli set as either 250 ms, 500 ms, or 750 ms. Under the alternate condition, time interval of the two auditory stimuli set to 1000 ms (Table 1).

**Table 1:** Tapping conditions and task characteristics

No.	Conditions	Tapping rate	Time interval of the two auditory stimuli	Tapping sets (each hand)
1	Simultaneous	2000 ms	0	20 sets
2	Gallop (250 ms)	2000 ms	250 ms	20 sets
3	Gallop (500 ms)	2000 ms	500 ms	20 sets
4	Gallop (750 ms)	2000 ms	750 ms	20 sets
5	Alternate	2000 ms	1000 ms	20 sets

Each trial started three seconds after the prompt “*Get ready!*” was displayed on the screen of the laptop PC. Participants completed a total of 40 taps per trial, with 20 taps per hand. This exclusion was based on the marked increase in variability observed in the beginning and end of each trial, particularly in the iNPH group. To control for possible edge effects, the first and last five taps in all groups were excluded from all analyses [29]. As a result, we analyzed the remaining 10 taps made with each hand (i.e., a total of 20 taps). The tapping rate of each hand was set to be 0.5 Hz (i.e., tapping every 2000 ms). Participants performed the task twice under each of the five tapping conditions (i.e., a total of 10 trials for the five conditions). For all but the simultaneous conditions, participants started the tapping with the right index finger in one trial and with the left index finger in the subsequent trial. Two different orders were employed to perform the five tapping conditions: (a) simultaneous, gallop (250 ms), gallop (500 ms), gallop (750 ms), and alternate conditions, and (b) alternate, gallop (750 ms), gallop (500 ms), gallop (250 ms), and simultaneous conditions. Half of the participants were assigned to perform the task in one of the two orders.

After completing the bimanual finger-tapping task, participants in the iNPH and older groups performed the FAB test to examine the participant’s frontal-lobe functions at the behavioral level [19, 28]. The test consists of six items: verbal conceptualization (manipulation of concepts by language), verbal fluency (intellectual flexibility), motor programming (function of higher motor cortex), sensitivity to interference (sensitivity to interference stimuli), inhibitory control (response inhibition), and environmental autonomy (prehension behavior). The FAB test is reasonably concise and does not require special equipment. A full score was 18; lower scores represented lower frontal lobe functions or cognitive functions [8].

### Data analysis

**Fig. 2:**
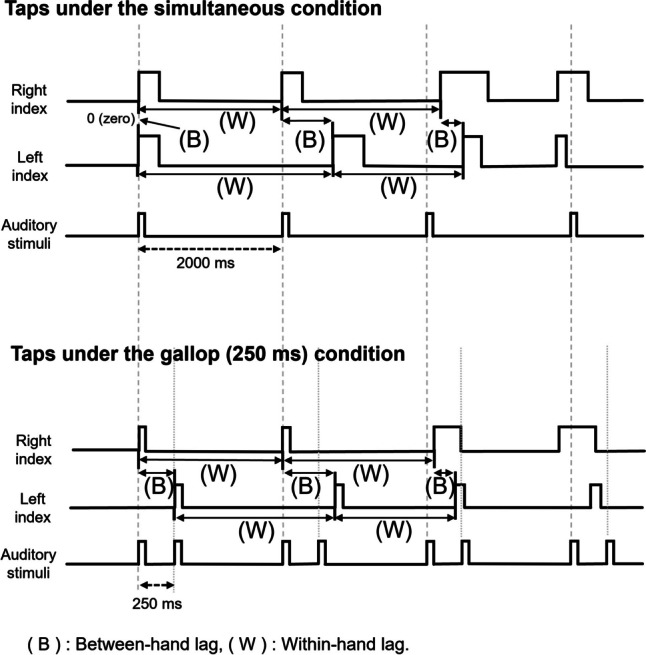
Temporal relationships between the audio stimuli and tap onsets under the two experimental conditions

Bimanual finger-tapping performance was described in terms of within-hand lag and between-hand lag (Fig. 2). Within-hand lag, the time interval between the two taps made by the same finger, represents the stability of single-handed movement during the bimanual coordination task [14, 17]. Since the interval between the two auditory stimuli presented for the same fingers was 2000 ms, within-hand lag indicated how far the tapping interval was from 2000 ms. As the within-hand lag was obtained from both fingers, we used the averaged data of the two values as dependent measures. The between-hand lag represented an inconsistency of the interval between the taps of the two fingers made in each tapping cycle (i.e., two taps in 2000 ms) [2, 9].

We assessed the within-hand lag and between-hand lag using three timing errors: variable error (VE), which measures intraparticipant variability; absolute error (AE) defined as the absolute value of deviation from the true lag; and constant error (CE), which quantifies the direction and magnitude of the deviation [33]. Each error type was calculated using the following equations [33]:

$$\begin{array}{l}\:VE=\sqrt{{\frac1n{\sum\:}_{i=1}^n(X_i-\overset-X)}^2},\\\:AE=\:\frac1n{\sum\:}_{i=1}^n\vert X_i-T\vert,\;\text{and}\\\:CE=\frac1n{\sum\:}_{i=1}^n(X_i-T),\end{array}$$

where $\:{{\rm\:X}}_{i}$ represents the interval measured in each cycle (ms); $\:\stackrel{-}{{\rm\:X}}$ is the average of all cycles (ms); $\:n$ is 20, corresponding to the number of tapping sets for analysis; $\:T$ represents the target interval (ms), i.e., 2000 ms for the within-hand lag, and either 0 ms, 250 ms, 500 ms, 750 ms, or 1000 ms for the between-hand lag. Lower VEs and AEs indicated better performance. The positive value of CE indicated that tapping performance was slower than the target interval.

### Statistical analysis

For each of the error types (i.e., VE, AE, and CE), a two-way ANOVA with repeated measures on the tapping condition was performed, factoring in the three groups and five tapping conditions. Significant main effects and interactions were further analyzed using Bonferroni-corrected pairwise comparisons. The significance level was set to 0.05%. Partial eta-squared values ($\:{\eta\:}_{\rho\:}^{2}$) were calculated to provide an unbiased estimate of effect size in the ANOVA. For each of six items in the FAB scores, an independent t-test was performed (iNPH group vs. older group). We also conducted one-way ANOVAs to test the effect of differences in age among the three groups and the χ^2^ test (chi-squared test) to test for the sex ratio.

As we did not calculate the statistical sample size for the study, we examined its adequacy by performing the power analysis (1 − $\:{\upbeta\:}$ err prob). This analysis considered the main results based on a significance level ($\:{\upalpha\:}$ = 0.05), effect size ($\:{\eta\:}_{\rho\:}^{2}$), and the number of participants (51 participants in total) using the G∗power software (Heinrich Heine University, Duesseldorf, Germany). The acceptable power level was set to 80.0% [7].

## Results

### Age and sex ratio

The analysis of age differences among the groups via ANOVA revealed a significant main effect of group ($\:F$(2, 48) = 230.76, *p* < 0.05, $\:{\eta\:}_{\rho\:}^{2}$ = 0.91). Multiple comparisons indicated that age was significantly greater in the iNPH and the older groups compared to the younger group, with no significant difference between the iNPH and older groups. The *post-hoc* power analysis (1 − $\:{\upbeta\:}$ err prob) for this effect yielded a power of 1.00. For the sex distribution, chi-square analysis did not show a significant difference between the groups ($\:{\chi\:}^{2}$ = 0.96, $\:p$ = 0.62, $\:V$ = 0.14). The *post-hoc* power analysis (1 − $\:{\upbeta\:}$ err prob) for this test indicated a power of 0.42, suggesting insufficient power to detect a significant effect, if present.

### Within-hand lag

**Fig. 3:**
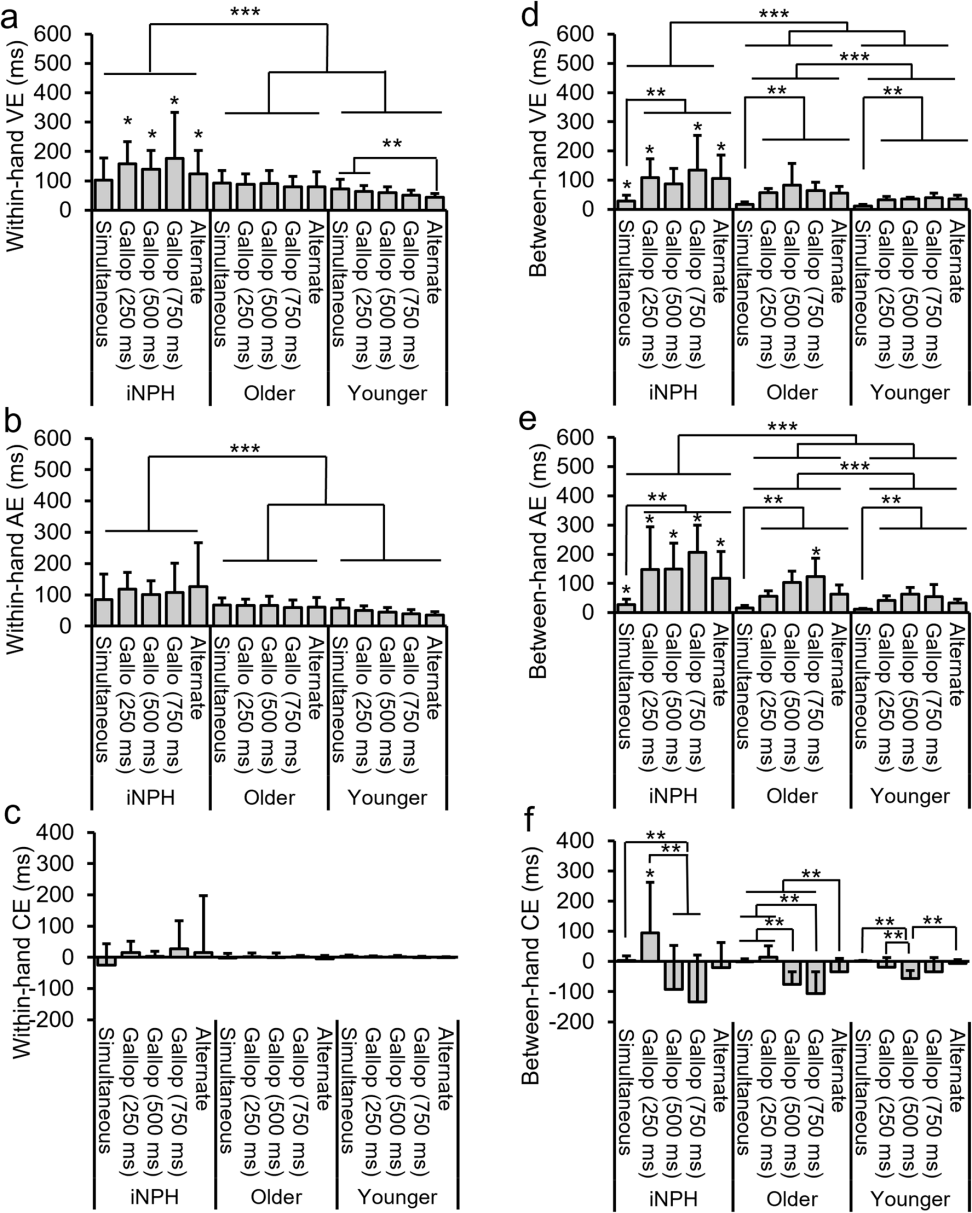
Results for each error type in within-hand lag and between-hand lag. **a**–**c** show the results for each of the Within-hand lag errors, and (**d**)–(**f**) show the Between-hand lag errors. For each tapping condition, only results of multiple comparisons are illustrated. ***: simple main effect tests between groups **: multiple comparisons within each group for different tapping conditions *: multiple comparisons for the interaction between groups and tapping conditions

The VE, AE, and CE for the within-hand lag across the five tapping conditions in each experimental group are shown in Fig. 3a, b, and c, respectively.

For VEs, the main effect of group was significant (*F*(2, 48) = 12.50, *p* < 0.05, $\:{\eta\:}_{\rho\:}^{2}$ = 0.34). Multiple comparisons indicated that VE was significantly higher in the iNPH group compared to the other two groups. The main effect of tapping condition, however, was not significantly different (*F*(4, 192) = 1.42, *p* = 0.23, $\:{\eta\:}_{\rho\:}^{2}$ = 0.03). There was a significant interaction between group and tapping condition (*F*(8, 192) = 2.40, *p* < 0.05, $\:{\eta\:}_{\rho\:}^{2}$ = 0.09), with further multiple comparisons showing that VE was significantly elevated in the iNPH group under the gallop (250 ms, 500 ms, 750 ms) and alternate conditions. In contrast, for the younger group, VE was significantly higher under the simultaneous and gallop (250 ms) conditions compared to the alternate condition. For *post-hoc* power analysis, the power (1 − $\:{\upbeta\:}$ err prob) for the main effect of group, the main effect of tapping conditions, and interaction were 1.00, 0.89, and 1.00, respectively.

For AE, the analysis revealed a significant main effect of group (*F*(2, 48) = 9.29, *p* < 0.05, $\:{\eta\:}_{\rho\:}^{2}$ = 0.28), indicating that AE was significantly greater in the iNPH group compared to the other two groups. The main effect of tapping condition did not show significant differences (*F*(4, 192) = 0.32, *p* = 0.86, $\:{\eta\:}_{\rho\:}^{2}$ = 0.006). There was no significant interaction between group and tapping condition (*F*(8, 192) = 1.26, *p* = 0.26, $\:{\eta\:}_{\rho\:}^{2}$ = 0.05). P*ost-hoc* power analysis (1 − $\:{\upbeta\:}$ err prob) showed a power of 1.00 for the main effect of group, 0.27 for the main effect of tapping conditions, and 0.97 for the interaction.

For CEs, neither the main effect of group (*F*(2, 48) = 1.07, *p* = 0.35, $\:{\eta\:}_{\rho\:}^{2}$ = 0.04), the main effect of tapping condition (*F*(4, 192) = 0.42, *p* = 0.79, $\:{\eta\:}_{\rho\:}^{2}$ = 0.008), nor the interaction (*F*(8, 192) = 0.53, *p* = 0.84, $\:{\eta\:}_{\rho\:}^{2}$ = 0.02) were significant. The *post-hoc* power analysis (1 − $\:{\upbeta\:}$ err prob) indicated a power of 0.42 for the main effect of group, 0.35 for the main effect of tapping condition, and 0.97 for the interaction.

### Between-hand lag

The VE, AE, and CE for the between-hand lag of the five tapping conditions in each experimental group are shown in Fig. 3d, e, and **f**, respectively.

For VE, a significant main effect of group was observed (*F*(2, 48) $\:=$ 19.13, *p* < 0.05, $\:{\eta\:}_{\rho\:}^{2}$ = 0.44), indicating that VE was significantly higher in the iNPH group compared to the other two groups. VE was also significantly higher in the older group than in the younger group and the main effect of tapping condition was significant (*F*(4, 192) = 11.74, *p* < 0.05, $\:{\eta\:}_{\rho\:}^{2}$ = 0.20), with VE being higher under the simultaneous condition than under the other four tapping conditions. There was a significant interaction between group and tapping condition (*F*(8, 192) = 2.11, *p* < 0.05, $\:{\eta\:}_{\rho\:}^{2}$ = 0.08), with further analysis revealing that VE was significantly greater in the iNPH group than in the other two groups under the simultaneous, gallop (250 ms), gallop (750 ms) and alternate conditions. *Post-hoc* power analysis confirmed a high statistical power (1 − $\:{\upbeta\:}$ err prob) for the main effect of group, the main effects of tapping conditions, and interaction, all of which were 1.00.

For AEs, a significant main effect of group was observed (*F*(2, 48) = 23.18, *p* < 0.05, $\:{\eta\:}_{\rho\:}^{2}$ = 0.49). Multiple comparisons showed that AE was significantly higher in the iNPH group compared to the other two groups. Further, AE was also significantly higher in the older group than in the younger group. The main effect of tapping condition was significant (*F*(4, 192) = 23.59, *p* < 0.05, $\:{\eta\:}_{\rho\:}^{2}$ = 0.33). Multiple comparisons showed that (a) AE was significantly greater under the simultaneous condition than under the other four conditions, (b) AE was significantly greater under the gallop (500 ms) and gallop (750 ms) conditions than under the alternate condition, and (c) AE was also significantly greater under the gallop (750 ms) condition than under the gallop (250 ms) condition. There was a significant interaction between group and tapping condition (*F*(8, 192) = 2.80, *p* < 0.05, $\:{\eta\:}_{\rho\:}^{2}$ = 0.10). Multiple comparisons showed that AE was significantly higher in the iNPH group compared to the other two groups under the simultaneous, gallop (250 ms), gallop (500 ms), and alternate conditions. Further, AE was also significantly greater in the iNPH and older groups compared to the younger group under the gallop (750 ms) tapping condition. For *post-hoc* power analysis, the power (1 − $\:{\upbeta\:}$ err prob) for the main effects of group, the main effects of tapping condition, and interaction were all 1.00.

For CE, the main effect of group was not significant (*F*(2, 48) = 0.37, *p* = 0.70, $\:{\eta\:}_{\rho\:}^{2}$ = 0.01). However, the main effect of tapping condition was significant (*F*(4, 192) = 26.18, *p* < 0.05, $\:{\eta\:}_{\rho\:}^{2}$ = 0.35). Multiple comparisons showed that CE was significantly greater under the simultaneous and gallop (250 ms) conditions compared to the other three conditions, and CE was also significantly higher under the alternate condition than under the gallop (500 ms) and gallop (750 ms) conditions. There was a significant interaction between group and tapping condition (*F*(8, 192) = 4.65, *p* < 0.05, $\:{\eta\:}_{\rho\:}^{2}$ = 0.16). Multiple comparisons showed that CE was significantly greater in the younger group than the other two groups under the gallop (250 ms) condition. The CE was also significantly higher in the iNPH group than the younger group under the gallop (750 ms) condition. For *post-hoc* power analysis, the power (1 − $\:{\upbeta\:}$ err prob) for the main effects of group, the main effects of tapping condition, and interaction were 0.17, 1.00, and 1.00, respectively.

### FAB scores

The FAB scores were significantly different (*p* < 0.05), with the iNPH group (mean score 12.31 ± 2.39) having lower scores than the older group (mean score = 16.80 ± 1.50).

## Discussion

The present study investigated whether patients with iNPH would encounter difficulties performing a bimanual finger-tapping task, particularly under the alternate tapping condition. The results showed that the VE for the within-hand lag in the iNPH group was significantly greater than in the other two groups, not only under the alternate condition, but also under the gallop (250 ms), gallop (500 ms), and gallop (750 ms) conditions (Fig. 3a). Notably, VE differences were not significant between the two control groups, suggesting that these variations were not attributable to aging alone. Our findings were consistent with those of a previous study [43] which also reported increased VE in the iNPH group under similar conditions. Additionally, the results obtained for CE in the present study align with previous findings [43], showing no significant differences across groups (Fig. 3c, f). Conversely, unlike the findings reported by Umemori [43], we observed no significant differences in AE under the alternate condition (Fig. 3b). Taken together, these findings support the idea that, at least in part, evaluating temporal errors in a bimanual coordination task, particularly in terms of within-hand coordination, is potentially useful to uncover pathological characteristics specific to iNPH.

For between-hand lag, both VE and AE were significantly greater in the iNPH group than in the other two groups across all five tapping conditions (Fig. 3d, e). This finding suggests that patients with iNPH have difficulty with bimanual coordination. Given the absence of significant differences in VE of within-hand lag among the groups under the simultaneous condition (Fig. 3a), patients with iNPH likely experienced more difficulty in maintaining consistent timing between hands, especially during asynchronous tapping tasks. This observation was consistent with our hypothesis that asynchronous bimanual coordination is particularly difficult for patients with iNPH and impaired SMA blood flow [35]. We confirmed that the iNPH group scored significantly lower on the FAB test than the older group, suggesting that patients with iNPH have declining frontal lobe function. Notably, although we expected patients with iNPH to show particular difficulty under the alternate condition, the results showed that their difficulties were also observed under other conditions. Based on these findings, we consider that the impaired frontal lobe functions of patients with iNPH resulted in them exhibiting difficulty in performing bimanual coordination, not only under isochronous (alternate) conditions, but also under the non-isochronous (gallop) conditions.

The results showed that VE and AE for within-hand lags did not show significant differences between the older and younger groups (Fig. 3a, b), suggesting that the within-hand coordination may not be significantly affected by aging. In contrast, VE and AE for between-hand lags were significantly greater in the older group compared to the younger group (Fig. 3d, e). These findings suggest that evaluating the quality of bimanual coordination, particularly in terms of the within-hand coordination, would be particularly useful for assessing pathological characteristics specific to iNPH, because within-hand coordination does not seem to be affected by age.

The findings partly replicated least some of the key results of Umemori et al. [43]. Firstly, the VE for within-hand lag under the alternate condition was significantly greater in the iNPH group than in the other two groups (Fig. 3a). Secondly, there were no significant differences in VE for within-hand lag under the simultaneous condition among the groups. Thirdly, no significant differences were observed in CEs for both within- and between-hand lags, indicating no directional trend in the temporal errors in the iNPH group (Fig. 3c, f). The consistency in these findings with those reported by Umemori et al. [43] underscores the robustness and reliability of the observed difficulties in performing the alternate condition of the bimanual finger-tapping task among patients with iNPH.

Notably, we also found several notable discrepancies with the findings of Umemori et al. [43]. In the present study, the AE of the within-hand lag was not significantly greater in the iNPH group than in the other groups under the alternate condition (Fig. 3b). This divergence from Umemori et al. [43] could potentially be attributed to the difference in the task setting between the two studies. The auditory stimuli in our study were presented at a frequency of 0.5 Hz under the simultaneous condition, while Umemori et al. [43] used a frequency of 1 Hz. The slower tempo used in this study may have made the tapping task more difficult than in Umemori et al. [43]. Indeed, Repp & Su [32] demonstrated that when the tempo is slower than 0.55 Hz, participants’ tapping performance to auditory stimuli tend to shift from an anticipatory to a reactive manner. This could make the tapping performance more difficult, even for young and older participants, and may have led to greater AE for the within-hand lag under the simultaneous condition. We speculate that this resulted in smaller differences between the groups under the simultaneous condition in the present study.

This study had three main limitations. Firstly, this study focused on bimanual coordination and did not adequately examine whether similar difficulties are encountered in the unimanual-alternate condition (e.g., alternating with the index and middle fingers). Consequently, it remains uncertain whether patients with iNPH have difficulty with the alternate condition itself or with the bimanual-alternate condition in particular. Future studies should include assessments of unimanual-alternate tasks condition to gain a more detailed understanding of the characteristics of bimanual coordination in patients with iNPH. Secondly, although participants with iNPH exhibited altered brain morphology, as indicated by DESH findings, cerebral blood flow and other activity levels were not assessed. Therefore, it remains unclear which specific areas of brain functionality are associated with the alternate condition. Future studies should employ neuroimaging techniques such as functional magnetic resonance imaging (fMRI) and single photon emission computed tomography (SPECT) to investigate the relationship between the alternate condition and activity in the SMA and other brain areas. Thirdly, our study did not implement age-matching between the iNPH group and the older group. While statistical analyses confirmed that there were no significant differences in age and sex between these groups, a case-controlled study with age-matching is necessary to confirm the robustness of the findings.

In conclusion, the findings of the present study suggest that patients with iNPH experience difficulties in bimanual coordination due impaired frontal lobe functions, observable not only under isochronous (alternate) conditions, but also under non-isochronous (gallop) conditions. Importantly, the absence of age-related impairments in within-hand lag (i.e., reflecting the stability of single-hand movement during bimanual tasks), suggests that the variability in within-hand lag is likely a pathological characteristic that is specific to iNPH. These findings underscore the need for further research to explore the neural mechanisms underlying these coordination challenges and to develop targeted interventions that address the unique motor deficits associated with this condition.

## Data Availability

The datasets analyzed during the current study are available from the corresponding author on reasonable request.

## Abbreviations

iNPH: Idiopathic normal pressure hydrocephalus
rTMS: Repetitive transcranial magnetic stimulation
SMA: Supplementary motor area
FAB: Frontal Assessment Battery
CSF: Subarachnoid cerebrospinal fluid
DESH: Disproportionately enlarged subarachnoid-space hydrocephalus
fMRI: functional magnetic resonance imaging
SPECT: single photon emission computed tomography
